# Emergency department use during COVID-19 as described by syndromic surveillance

**DOI:** 10.1136/emermed-2020-209980

**Published:** 2020-09-18

**Authors:** Helen E Hughes, Thomas C Hughes, Roger Morbey, Kirsty Challen, Isabel Oliver, Gillian E Smith, Alex J Elliot

**Affiliations:** 1 Real-time Syndromic Surveillance Team, Public Health England, Birmingham, UK; 2 University of Liverpool Faculty of Health and Life Sciences, Liverpool, UK; 3 John Radcliffe Hospital, Oxford, UK; 4 Lancashire Teaching Hospitals NHS Foundation Trust, Chorley, UK; 5 Field Service, National Infection Service, Public Health England, Bristol, UK

**Keywords:** emergency department utilisation, infectious diseases, viral, epidemiology

## Abstract

On 12 March 2020 the UK entered the ‘delay phase’ of the COVID-19 pandemic response. The Public Health England Emergency Department Syndromic Surveillance System (EDSSS) carries out daily (near real-time) public health surveillance of emergency department (ED) attendances across England. This retrospective observational analysis of EDSSS data aimed to describe changes in ED attendances during March–April 2020, and identify the attendance types with the largest impact. Type 1 ED attendances were selected from 109 EDs that reported data to EDSSS for the period 1 January 2019 to 26 April 2020. The daily numbers of attendances were plotted by age group and acuity of presentation. The 2020 ’COVID-19’ period (12 March 2020 to 26 April 2020) attendances were compared with the equivalent 2019 ’pre-COVID-19’ period (14 March 2019 to 28 April 2019): in total; by hour and day of the week; age group(<1, 1-4, 15-14, 15-44, 45-64 and 65+ years); gender; acuity; and for selected syndromic indicators(acute respiratory infection, gastroenteritis, myocardial ischaemia). Daily ED attendances up to 11 March 2020 showed regular trends, highest on a Monday and reduced in children during school holidays. From 12 March 2020 ED attendances decreased across all age groups, all acuity levels, on all days and times. Across age groups the greatest percentage reductions were seen in school age children (5–14 years). By acuity, the greatest reduction occurred in the less severe presentations. Syndromic indicators showed that the greatest reductions were in non-respiratory indicators, which fell by 44–67% during 2020 COVID-19, while acute respiratory infection was reduced by −4.4% (95% CI −9.5% to 0.6%). ED attendances in England have been particularly affected during the COVID-19 pandemic due to changes in healthcare seeking behaviour. EDSSS has enabled real-time daily monitoring of these changes, which are made publicly available to facilitate action. The EDSSS provides valuable surveillance of ED attendances in England. The flexibility of EDSSS allowed rapid development of new indicators (including COVID-19-like) and reporting methods.

## Background

The COVID-19 pandemic has had major health and societal impacts worldwide. In the UK, the ‘delay phase’ was introduced in stages from 12 March 2020, including social distancing and shielding measures.^1^ These have had a major impact on population movement, day-to-day activities and healthcare seeking behaviours.

The Public Health England (PHE) Emergency Department Syndromic Surveillance System (EDSSS) is a public health legacy of the London Olympic and Paralympic Games 2012, receiving routine data from emergency departments (EDs) across England, captured through the Emergency Care Dataset (ECDS).^2–4^ This anonymised subset of ECDS data is received on a daily basis, enabling a near real-time syndromic surveillance service, which feeds into PHE public health monitoring activities (including the COVID-19 response) and with weekly EDSSS surveillance bulletins made publicly available.^5 6^ The EDSSS is an unvalidated ‘snapshot’ of raw ED data (updates or completion of missing data are not included), which can be used for timely analysis and identification of trends for public health purposes.

In this short report we use routine EDSSS data to describe the changes in ED attendances in England from 12 March 2020, and the subsequent challenges that this has brought to undertaking ED syndromic surveillance.

## Methods

### Attendance data

Daily ED attendance data were accessed from EDSSS from 1 January 2019 to 26 April 2020 (routine, anonymised, public health surveillance data, no ethical approval required). Selection criteria for inclusion were: type 1 ED attendances; EDs reporting attendances for every day during the study period. EDSSS includes only EDs located in England.

ED attendances categorised by syndromic indicator were identified based on the primary diagnosis listed for each attendance (if any). In this report, syndromic indicators routinely identified in EDSSS were acute respiratory infections, gastroenteritis and myocardial ischaemia.

### Descriptive analysis

Daily attendances were visualised by calendar years (2019 full year; 2020 to 26 April 2020), by age group (0, 1–4 and 15–14, 15–44, 45–64 and 65+years) and separately by acuity of attendance (ECDS values from 1 (immediate) to 5 (low acuity)).

Separate comparable time periods, matched on day of the week, were identified for the 2019 ‘pre-COVID-19’ period (Thursday 14 March 2019 to Sunday 28 April 2019) and the 2020 'COVID-19’ period during the delay phase (Thursday 12 March 2020 to Sunday 26 April 2020). The mean number of all-cause all-age attendances were plotted by hour of day and day of the week for both the pre-COVID-19 and COVID-19 periods.

The average daily attendances were calculated with the percentage difference between pre-COVID-19 and COVID-19 in total by sex, age group, acuity, day of the week and by selected syndromic surveillance indicators.

## Results

A total of 109 type 1 EDs met the inclusion criteria, reporting a total of 13 861 889 attendances to EDSSS from 1 January 2019 to 26 April 2020.

**Figure 1:**
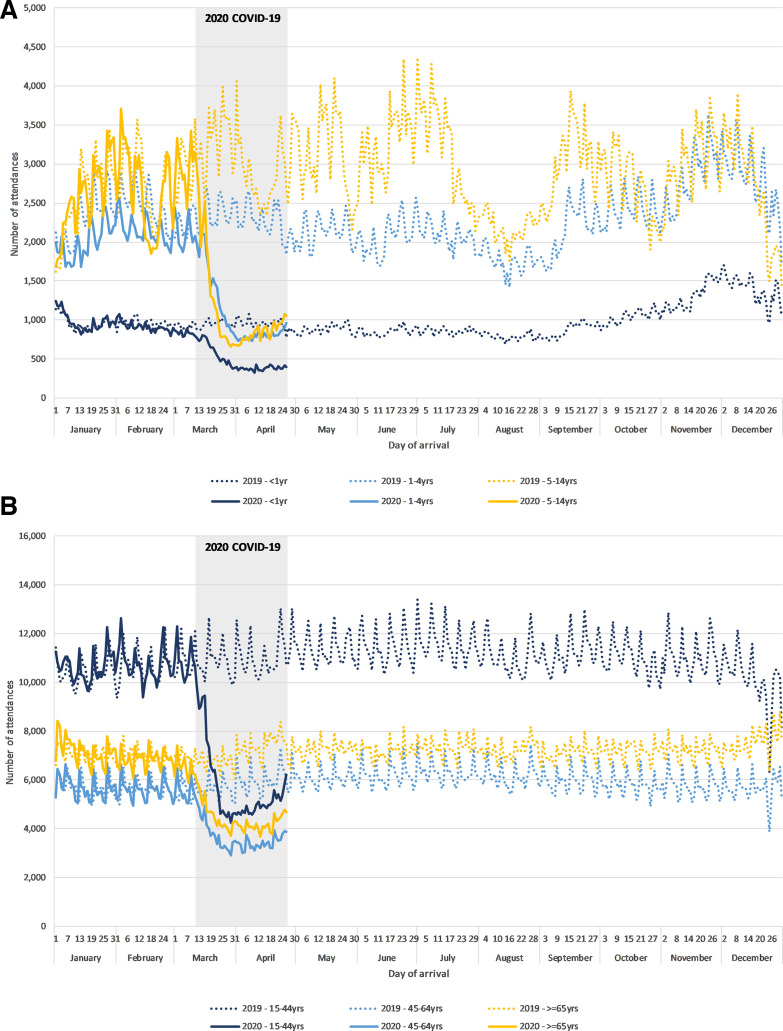
Daily Emergency Department Syndromic Surveillance System attendances in 2019 and 2020 by age group for (A) children and (B) adults (n=109 EDs). The 2020 COVID-19 period (12 March 2020 to 26 April 2020) is marked in grey.

Daily attendances by age group to 11 March 2020 showed similar trends—that is, peak attendances on Monday and a notable reduction in child attendances during school holidays. From 12 March 2020 the numbers of daily attendances rapidly decreased across all age groups (figure 1).

The largest percentage change reduction in attendances was in school age children (table 1). There was no clear difference by gender (table 1). Age and gender were reported for >99.5% of all attendances in both years.

**Table 1:** Differences in ED attendances between 2019 pre-COVID-19 and 2020 COVID-19 (based on the periods 14 March 2019 to 28 April 2019 and 12 March 2020 to 26 April 2020, respectively, matched on day of the week)

	2019 pre-COVID-19	2020 COVID-19	Percentage change (95% CI)
Total	30 412	16 217	−46.7% (−50.4% to −42.9%)
Age			
<1 year	954	471	−50.6% (−55.2% to −46.1%)
1–4 years	2318	1068	−53.9% (−59.4% to −48.5%)
5–14 years	3026	1075	−64.5% (−70.8% to −58.2%)
15–44 years	10 982	5636	−48.7% (−53.0% to −44.4%)
45–64 years	5842	3596	−38.5% (−42.0% to −34.9%)
≥65 years	7143	4360	−39.0% (−42.1% to −35.8%)
Gender			
Female	15 438	8171	−47.1% (−50.6% to −43.5%)
Male	14 959	8020	−46.4% (−50.4% to −42.4%)
Day of the week			
Monday	33 594	16 778	−50.1% (−59.3% to −40.9%)
Tuesday	31 394	15 627	−50.2% (−59.2% to −41.2%)
Wednesday	30 498	15 385	−49.6% (−55.4% to −43.7%)
Thursday	30 241	16 752	−44.6% (−55.3% to −33.9%)
Friday	29 682	16 456	−44.6% (−53.5% to −35.6%)
Saturday	28 675	16 358	−43.0% (−52.8% to −33.1%)
Sunday	29 410	16 042	−45.5% (−54.2% to −36.7%)
Acuity			
1: Immediate	370	256	−30.7% (−33.9% to −27.6%)
2: Very urgent	2336	1504	−35.6% (−38.2% to −33.0%)
3: Urgent	9416	5642	−40.1% (−43.5% to −36.7%)
4: Standard	11 818	5455	−53.8% (−58.0% to −49.7%)
5: Low acuity	1284	684	−46.7% (−54.3% to −39.0%)
Selected syndromic indicators			
Acute respiratory infections	1757	1679	−4.4% (−9.5% to 0.6%)
Gastroenteritis	356	118	−66.9% (−71.4% to −62.4%)
Myocardial ischaemia	357	199	−44.2% (−48.8% to −39.7%)

**Figure 2:**
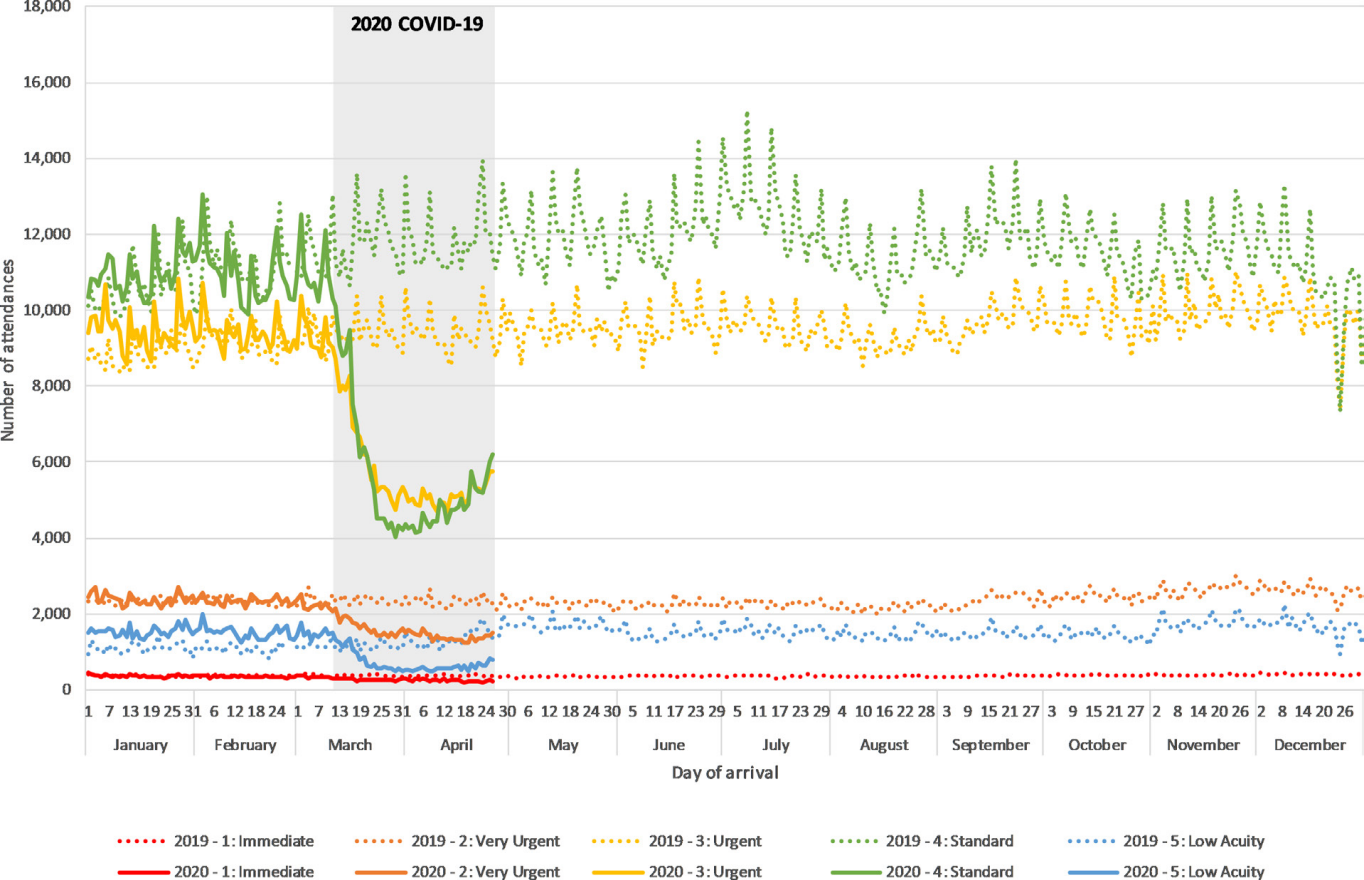
Daily Emergency Department Syndromic Surveillance System attendances in 2019 and 2020 by acuity, where known (n=109 EDs). The 2020 COVID-19 period (12 March 2020 to 26 April 2020) is marked in grey.

The level of acuity was identifiable in 83.6% of all attendances (82.9% pre-COVID-19; 83.5% COVID-19). Those with an acuity of ‘1: immediate’ accounted for the smallest numbers of ED attendances and saw the smallest reduction in levels during COVID-19 (31%), and those with an acuity of ‘4: Standard’ saw the largest reduction (54%; table 1 and figure 2).

**Figure 3:**
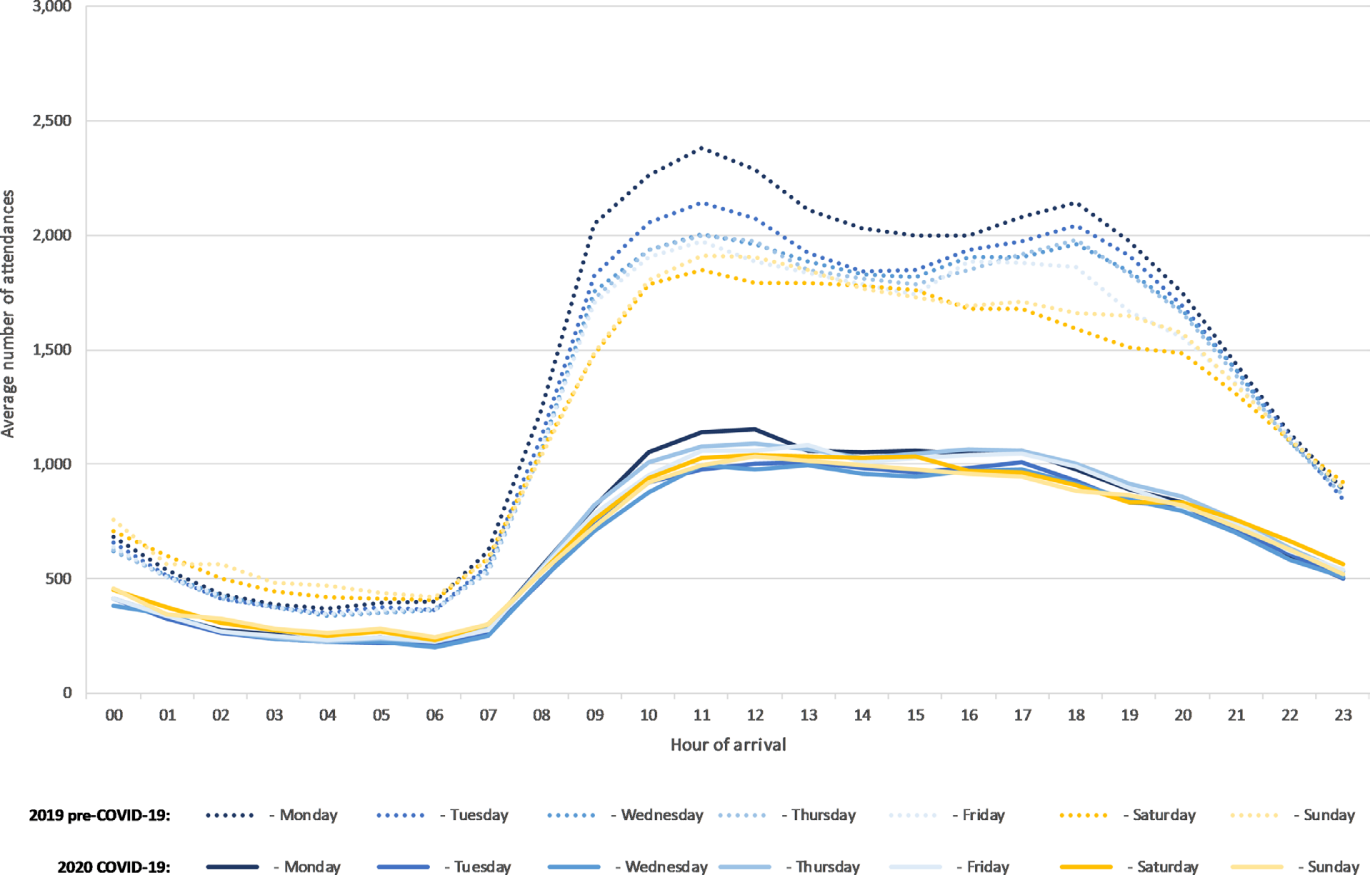
Emergency Department Syndromic Surveillance System attendances by hour of day and day of week during the 2019 pre-COVID-19 and 2020 COVID-19 periods (based on the periods 14 March 2019 to 28 April 2019 and 12 March 2020 to 26 April 2020, respectively, matched on day of the week).

Attendance levels were reduced throughout the 24-hour period (figure 3). The largest decrease was seen on Monday to Wednesday, previously the busiest days of the week (table 1).

Syndromic indicators showed that the greatest reductions were in non-respiratory indicators. While there was only a 4% reduction in acute respiratory infections, non-respiratory indicators fell by 44–67% during COVID-19 (table 1).

## Discussion

During the 2020 COVID-19 period there were fewer daily ED attendances than in the 2019 pre-COVID-19 period. The largest percentage reductions were observed on Monday to Wednesday (previously the busiest days of the week) and in the youngest age groups (particularly school age children). The reduction was observed across all acuity categories, although it was less marked in the most severe attendance presentations. These findings support and quantify a recent Royal College of Emergency Medicine position statement in the UK and also corroborate similar recent findings from the USA.^7 8^

EDSSS reports on high level groupings of disease/condition indicators which provide additional depth of understanding of ED activity, particularly with respect to infectious diseases. While other official sources of ED activity data in the UK (eg, the NHS England weekly and monthly admission statistics^9^) provide information about overall attendance activity, they include other service metrics such as patient wait times to inform performance management. Routine reporting of EDSSS data supplements these other sources and illustrates a differential impact of the changes in healthcare seeking behaviour (in real-time)—for example, attendances for acute respiratory infections decreased very little but non-respiratory indicators reported here decreased by 44–67%. Monitoring these changes in healthcare utilisation through surveillance is key to understanding the impact of COVID-19 in the population. These syndromic surveillance data demonstrate possible indirect impacts of social distancing/shielding, both positive (eg, reduced need for gastroenteritis attendances) and negative (eg, emergency cardiac care potentially avoided). Recent public health messaging has urged patients to continue to seek medical care as required.^10^

The routine nature of the EDSSS enabled the rapid comparison of pre- and current COVID-19 periods to describe the impact using a large subset of English type 1 ED attendances. However, this analysis is limited by the intentional exclusion of all non-type 1 ED attendances and some type 1 ED attendances due to inconsistency in the frequency of data submission. The intention is for NHS acute data to be submitted to NHS Digital, using ECDS, on a daily basis.^4^

One of the biggest challenges for EDSSS has been changes in the total attendances which led to difficulty interpreting syndromic indicators as a percentage of attendances, resulting in false signals. EDSSS reporting was subsequently rapidly adapted, with attendance counts (as used here) presented in all standard EDSSS reporting from 19 March 2020.^5^ Supplementary EDSSS developments will include severity indicators to provide enhanced intelligence in future.

The EDSSS now reports on COVID-19-like attendances (including new COVID-19 SNOMED codes^11^). This information now feeds into the PHE COVID-19 response, demonstrating that the information is actionable, as well as in regular weekly EDSSS surveillance bulletins.^5 6^ Furthermore, EDSSS outputs are also used by the UK Government to support and guide management of the pandemic. EDSSS will continue to be used during the COVID-19 pandemic, delivering real-time monitoring of indicators of both direct (respiratory) and indirect (non-respiratory) healthcare demand. It will also provide valuable surveillance information during any future waves and inform on healthcare pressures during winter 2020/21 when SARS-CoV-2 and other seasonal respiratory pathogens will impact on emergency care services.^12^

## Conclusion

ED attendances in England have been affected by changes in healthcare seeking behaviour during the COVID-19 pandemic. EDSSS has enabled real-time daily monitoring of these changes, providing publicly available information to facilitate action. The EDSSS provides valuable surveillance of ED attendances in England. The flexibility of EDSSS allowed rapid development of new indicators (including COVID-19-like) and changes to reporting methods as required.
